# Depression and Anxiety in Patients With Polycystic Ovary Syndrome: A Cross-Sectional Study in Saudi Arabia

**DOI:** 10.7759/cureus.51530

**Published:** 2024-01-02

**Authors:** Latteefah Alnaeem, Muntaha Alnasser, Yaqin AlAli, Fatimah Almarri, Abdulmuhsin A Al Sultan, Fatimah A Almuhaysin, Nadeen A Boubshait, Latifah A Almulhim

**Affiliations:** 1 Obstetrics and Gynaecology, King Faisal University, Al-Ahsa, SAU; 2 College of Medicine, King Faisal University, Al-Ahsa, SAU

**Keywords:** stress, middle east, polycystic ovary syndrome, anxiety, depression

## Abstract

Introduction: Polycystic ovary syndrome (PCOS) is the most common endocrine disorder in females of childbearing age. It causes irregular menstruation, infertility, acne vulgaris, androgenic alopecia, and hirsutism. It is associated with a higher risk of mental disorders. This study aimed to determine the prevalence of depression and anxiety among females with PCOS and the factors associated with these disorders.

Methods: This cross-sectional study was conducted between 15th January and 19th November 2023. We invited Saudi women to do an online survey. We sent the survey link privately, got their permission, and explained the research to ensure privacy and reliability. Females with a previous psychiatric history were excluded. Depression, Anxiety, and Stress Scale-21 Items (DASS-21) were used to assess depression, anxiety, and distress. One-way analysis of variance (ANOVA) and two-sample t-tests were used to identify determinants of depression and anxiety.

Results: About 967 females participated, of whom 474 (49%) were married, and 358 (37%) had a healthy weight. About 367 (37.9%) of participants were diagnosed with PCOS, and it was associated with age (26-35 years), divorce, and family history of PCOS (p < 0.05). About 112 (30.5%) of PCOS patients experienced extremely severe depression, and 144 (39.2%) had extreme anxiety. People in the age range of 15-25 years had a higher risk of depression and stress (p < 0.05). Divorced participants faced a higher risk of depression, anxiety, and stress than singles (p < 0.05). Those advised on diet and healthy lifestyles exhibited a higher risk of depression, anxiety, and stress than those who were not (p < 0.05). Additionally, being overweight was associated with a higher risk of depression (p < 0.05).

Conclusion: The prevalence of PCOS was found to be 37.9% in our study, which may seem higher compared to the existing literature on PCOS. It is associated with being in the age group of 26-35 years, being divorced, and having a positive family history. Almost two-thirds of females with PCOS had depression, anxiety, and stress. Factors associated with the three disorders include divorce and management with diet and lifestyle modifications. Depression and stress were associated with young age. High body mass index (BMI) was associated with depression.

## Introduction

Polycystic ovary syndrome (PCOS) is the most common endocrine disorder of females in the reproductive age group [1]. It is a multifaceted disease that causes metabolic, endocrine, and reproductive abnormalities [2]. A recent systematic review and meta-analysis reported that the pooled prevalence of PCOS was 21.27% [3]. This prevalence is higher than the 10%-13% prevalence confirmed in 2023 guidelines on diagnosing PCOS [2]. The difference may be attributed to the diagnostic criteria used. Based on the best available evidence, it is recommended to diagnose PCOS when at least two of the following exist: clinical or biochemical hyperandrogenism, ovulatory dysfunction, polycystic ovaries on ultrasound, or a high level of anti-Mullerian hormone (AMH) [2].

PCOS is associated with metabolic syndrome presenting as central obesity, increased fasting blood glucose, elevated blood pressure, increased triglyceride, increased low-density lipoprotein (LDL), and reduced high-density lipoprotein (HDL) [4]. PCOS also causes hyperandrogenism, leading to a higher concentration of testosterone [5]. It is associated with irregular menstruation, infertility, acne vulgaris, androgenic alopecia, and hirsutism [6].

No specific drug is approved by the United States Food and Drug Administration (USFDA) to manage PCOS [6]. The current recommendations include lifestyle changes, combined oral contraceptive pills for menstrual irregularity, and metformin for metabolic disturbances [2]. Letrozole, clomiphene, and in vitro fertilization (IVF) are used for infertility treatment [2].

Physical attractiveness is believed to determine success and quality of life in the twenty-first century [7,8]. Obesity, hirsutism, and acne of PCOS are far from attractive [7]. A qualitative study on females with PCOS found their main concern to be the threat of feminine identity caused by PCOS [9]. The feminine identity is not limited to physical attractiveness but includes fertility, which is essential for marital satisfaction [9,10]. These concerns may impair the psychological well-being of females with PCOS [11].

A study by Ghazeeri et al. reported a lack of association between PCOS, depression, and anxiety [12]. However, a recent meta-analysis found that PCOS is significantly associated with them [13]. Factors associated with the psychological burden of PCOS include rural residence, low education, high body mass index (BMI), being unmarried, and being childless [11,14-16]. These associations are not universal as some studies reported no association between BMI and education with depression and anxiety in females with PCOS [8]. This study aimed to determine the prevalence of anxiety, depression, and perceived stress in females with PCOS. It also aimed to identify the factors associated with the occurrence of these mental disorders in this group of females.

## Materials and methods

Study overview

This cross-sectional study aimed to investigate the prevalence and determinants of depression, anxiety, and stress among Saudi females diagnosed with PCOS.

Ethical considerations

Ethical approval for the study was obtained from the King Faisal University Ethical Committee (Reference: KFU-REC-2022-DEC-ETHICS398). Participants provided free and informed consent, ensuring anonymity and data privacy. The study adhered to the principles outlined in the Declaration of Helsinki.

Study criteria (study population)

The study targeted Saudi females with a confirmed diagnosis of PCOS. Participants were recruited through various social media platforms between January 15 and November 19. Before participation, individuals received a direct invitation, accompanied by an explanation of the research purpose to ensure confidentiality and reliability of responses.

Study procedure

An online self-administered questionnaire, distributed through private messages and emails, was employed for data collection. Permission was obtained from participants before their involvement. The questionnaire, divided into three sections, covered sociodemographic data, PCOS-related information, and the Arabic version of the Depression, Anxiety, and Stress Scale (DASS-21).

Assessments

The DASS-21 comprised 21 items, categorized into depression, anxiety, and stress. Respondents rated items on a four-point Likert scale. Subscale scores (depression, anxiety, and stress) were derived by multiplying the total points accumulated across the items by 2.

Sample size calculation

A minimal sample size of 385 was calculated using the unknown population formula (n = z^2^pq/d^2^), with parameters set at a 5% margin of error (d), 50% expected proportion (p), and a confidence level of 95% (z = 1.96). To account for the convenience sampling approach, 967 responses were collected.

Statistical analysis

Data cleaning was performed in Microsoft Excel, and statistical analysis was conducted using R software version 4.2.2. The normality of distribution was assessed using a histogram and Kolmogorov-Smirnov test. Descriptive statistics determined the mean and standard deviation for continuous variables, while frequencies and percentages represented categorical variables. One-way ANOVA, two-sample t-tests, Pearson's Chi-square, and Fisher's exact tests were employed to identify determinants and assess associations among PCOS participants. A significance level of 0.05 was set.

## Results

A total of 967 people participated in the study, with 461 (48%) aged 15-25 years. Approximately 474 (49%) were married, 941 (97%) were Saudi, and 580 (60%) resided in the northern region of Saudi Arabia. About 358 (37%) had a healthy weight, and 264 (27.3%) had 0-2 children (Table 1).

**Table 1:** Demographic characteristics of the participants ^1^n (%).

Characteristics	N = 967^1^
Age (years)	
15-25	461 (48%)
26-35	191 (20%)
36-45	155 (16%)
46 and above	160 (17%)
Marital status	
Single	444 (46%)
Married	474 (49%)
Divorced	27 (2.8%)
Widowed	22 (2.3%)
Nationality	
Non-Saudi	25 (2.6%)
Saudi	941 (97%)
Missing	1
Residence area	
Central	17 (1.8%)
Eastern	334 (35%)
Northern	580 (60%)
Southern	5 (0.5%)
Western	17 (1.8%)
Outside Saudi Arabia	13 (1.3%)
Missing	1
Body mass index	
Healthy weight	358 (37%)
Underweight	94 (9.8%)
Overweight	271 (28%)
Obese	240 (25%)
Missing	4
Number of children	
0-2	264 (27.3%)
3 and above	259 (26.7%)
Missing	444

**Figure 1:**
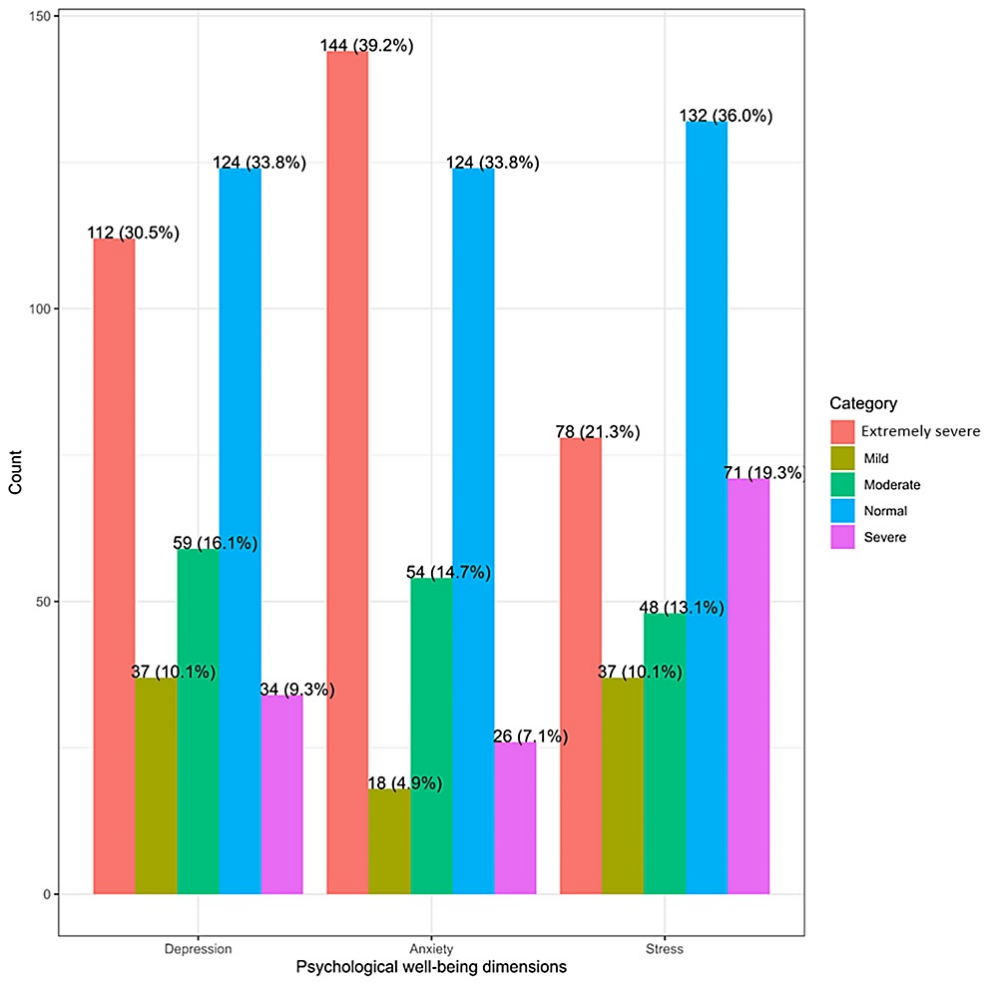
Psychological well-being dimensions

As shown in Figure 1, 37 (10.1%) respondents had mild depression, 59 (16.1%) had moderate depression, 34 (9.3%) had severe depression, and 112 (30.5%) had extremely severe depression. Similarly, 37 (10.1%) respondents had mild stress, 48 (13.1%) had moderate stress, 71 (19.3%) had severe stress, and 78 (21.3%) had extremely severe stress. Similarly, 18 (4.9%) respondents had mild anxiety, 54 (14.7%) had moderate anxiety, 26 (7.1%) had severe anxiety, and 144 (39.2%) had extremely severe anxiety.

Table 2 revealed that 31% had a family history, 69% were diagnosed with ultrasound, and 21% were diagnosed using blood tests, with 73% prescribed medication. The median age at diagnosis was 22 years.

**Table 2:** Characteristics of participants with polycystic ovary disease ^1^n (%); median (IQR). *Percentages may not sum to 100 as it is a multiple-choice question.

Characteristics	N = 367^1^
Do you have a family history of polycystic ovary disease?	
No	163 (44%)
Yes	114 (31%)
I don’t know	90 (25%)
Age at diagnosis	22 (18, 25)
Unknown	16
The diagnosis was based on	
Ultrasound	244 (69%)
Blood test	74 (21%)
I don’t know	36 (10%)
Unknown	13
Have you been prescribed treatment?	
Yes	258 (73%)
No	96 (27%)
Unknown	13
Treatment*	
Hormonal	155 (44%)
Metformin	132 (37%)
Diet and lifestyle modification	90 (25%)
I don’t use treatment	87 (25%)
Oral contraceptive	9 (2.5%)
Surgery	3 (0.8%)
Unknown	16

Table 3 ​​​presents the results of statistical analyses examining the association between participant characteristics and the prevalence of depression, anxiety, and stress in the context of PCOS. Notably, age cohorts showed significant variations in depression (p = 0.015) and stress levels (p = 0.014), with the 15-25 age group experiencing higher depression (19 ± 14) and stress (22 ± 13) compared to older age groups. Marital status exhibited notable associations across all three mental health parameters (depression: p = 0.001, anxiety: p = 0.004, stress: p = 0.016), indicating higher levels among divorced individuals (depression: 25 ± 14, anxiety: 20 ± 12, stress: 26 ± 12). Interestingly, the residence area did not significantly impact depression, anxiety, or stress levels although the central region reported elevated rates. Treatment methods also showed varying degrees of influence, with lifestyle changes indicating higher levels of depression (21 ± 13), anxiety (18 ± 12), and stress (23 ± 12) (depression: p = 0.015, anxiety: p = 0.019, stress: p = 0.022). BMI exhibited significant associations with depression (p = 0.010), showcasing higher rates among overweight individuals (21 ± 14). The table also provides insights into the relationships between mental health outcomes and other participant characteristics, including nationality, residence area, age at diagnosis, family history of polycystic ovary disease, treatment methods, diet and lifestyle changes, and the number of children. The presented mean scores for depression, anxiety, and stress offer a detailed understanding of the impact of these factors on the mental health of individuals with PCOS.

**Table 3:** Determinants of depression, anxiety, and stress among polycystic ovary disease participants ^1^ Mean ± SD. ^2^ One-way ANOVA; Welch's two-sample t-test.

	Depression		Anxiety		Stress	
Characteristics	N = 367^1^	p-value^2^	N = 367^1^	p-value^2^	N = 367^1^	p-value^2^
Age		0.015		0.2		0.014
15-25	19 ± 14		16 ± 12		22 ± 13	
26-35	17 ± 13		14 ± 11		20 ± 13	
36-45	18 ± 14		17 ± 12		20 ± 13	
46 and above	10 ± 11		11 ± 13		13 ± 13	
Nationality		0.6		0.3		0.4
Non-Saudi	20 ± 13		12 ± 13		18 ± 11	
Saudi	18 ± 14		15 ± 12		21 ± 13	
Unknown	1		1		1	
Residence area		0.2		0.3		0.6
Central	28 ± 15		22 ± 10		27 ± 10	
Northern	18 ± 14		15 ± 12		21 ± 13	
Southern	21 ± 8		17 ± 11		23 ± 8	
Western	18 ± 13		17 ± 10		22 ± 12	
Eastern	18 ± 13		15 ± 12		20 ± 13	
Outside Saudi Arabia	20 ± 14		10 ± 13		18 ± 11	
Marital status		0.001		0.004		0.016
Single	20 ± 14		17 ± 12		22 ± 13	
Divorced	25 ± 14		20 ± 12		26 ± 12	
Married	15 ± 13		13 ± 11		19 ± 13	
Age at diagnosis		0.14		0.14		0.055
12-20	19 ± 14		15 ± 13		21 ± 13	
21-30	19 ± 13		16 ± 12		21 ± 13	
31 and above	14 ± 13		12 ± 12		16 ± 14	
Unknown	16		16		16	
Do you have a family history of polycystic ovary disease?		0.9		0.8		0.8
No	18 ± 14		15 ± 13		21 ± 13	
Yes	18 ± 14		16 ± 12		20 ± 14	
I don’t know	19 ± 13		15 ± 11		21 ± 11	
Treatment						
Metformin		0.075		0.2		0.081
No	17 ± 13		15 ± 12		20 ± 13	
Yes	20 ± 14		16 ± 13		22 ± 13	
Unknown	14		14		14	
Oral contraceptive		0.14		0.14		0.13
No	18 ± 14		15 ± 12		20 ± 13	
Yes	23 ± 10		21 ± 10		28 ± 13	
Unknown	14		14		14	
Surgery		>0.9		0.5		0.5
No	18 ± 14		15 ± 12		21 ± 13	
Yes	17 ± 14		21 ± 15		26 ± 11	
Unknown	14		14		14	
Hormonal		0.9		0.5		>0.9
No	18 ± 14		15 ± 12		21 ± 13	
Yes	18 ± 14		16 ± 12		21 ± 13	
Unknown	14		14		14	
Diet and lifestyle change		0.015		0.019		0.022
No	17 ± 14		14 ± 12		20 ± 13	
Yes	21 ± 13		18 ± 12		23 ± 12	
Unknown	14		14		14	
Number of children		0.5		0.3		0.5
0-2	16 ± 13		13 ± 11		20 ± 13	
3 and above	15 ± 13		15 ± 12		18 ± 13	
Unknown	186		186		186	
BMI		0.010		0.073		0.15
Underweight	13 ± 12		12 ± 11		17 ± 12	
Healthy weight	16 ± 13		14 ± 12		20 ± 13	
Overweight	21 ± 14		17 ± 12		22 ± 13	
Obese	19 ± 13		16 ± 12		21 ± 13	
Unknown	2		2		2	

## Discussion

This study used validated tools to assess the prevalence of psychiatric disorders among females with PCOS compared to healthy females. It also reported the factors associated with various mental disorders. In this study, depression, anxiety, and stress were present in almost two-thirds of participants. Factors associated with the three disorders include divorce and management with diet and lifestyle modifications. Depression was also associated with high BMI and young age. Anxiety was associated with young age.

In our cohort, most females with a clinical diagnosis of PCOS were in the age groups of 15-25 and 26-35. Among those with PCOS, the median age of diagnosis was 22 years. This is slightly younger than the age at diagnosis in the United States (26.9 years) [17]. A previous study reported that PCOS diagnosis is associated with extremes of symptoms and fertility concerns, which encouraged contact with health professionals [18]. Interestingly, PCOS was associated with divorce in this cohort of participants. In the Middle East, females are expected to get pregnant soon after marriage; a qualitative study from Oman, which is culturally similar to Saudi Arabia, revealed that females were blamed for infertility, and in-laws suggested second marriage or even divorce, with threats being more apparent as the infertility duration increases [19]. It should be acknowledged that more than a third of married females had PCOS, and almost half of the females with PCOS were married. Though it affects fertility, PCOS does not imply being childless. Both medical, like clomiphene citrate, and surgical interventions, like in vitro fertilization, can be used to treat infertility in females with PCOS [2].

BMI was not associated with PCOS in our participants. More than half of the participants were overweight or obese, consistent with the high prevalence of obesity in the general population of Saudi Arabia, which is reported to be 35.6% [20]. We speculate that the high prevalence of obesity masked the association consistently reported in the literature [4,21]. In our cohort, 358 (37%) of females with PCOS were of healthy weight, and 27 (7.4%) were underweight. Despite the association between high BMI and PCOS, it should be remembered that being overweight is not a prerequisite to PCOS diagnosis, and healthy weight as well as underweight cases are noted [22,23]. The self-reported nature of BMI should be kept in mind when interpreting these results. A family history of PCOS was significantly associated with a higher risk of developing PCOS. A similar finding is noted in Egypt [24]. The level of anti-Mullerian hormone is higher in girls with premature adrenarche whose mothers had PCOS, and the high anti-Mullerian hormone is believed to increase their risk of PCOS [25].

When asked about their treatment, only a quarter of females reported diet and lifestyle modifications. This is concerning as a healthy lifestyle is the first step in managing PCOS and should be encouraged throughout the lifespan of the patients [2]. Of note, a quarter of females who reported a diagnosis of PCOS claimed that they used no treatment. Most drugs used in the management of PCOS are off-label, and the relatively limited evidence should be acknowledged in practice [2]. It is possible that females who used no therapy had mild symptoms, and weighing the risks with benefits resulted in this decision. However, they should have at least been advised on following a healthy lifestyle. Future studies should address physicians' approach to PCOS management and investigate why they did not advise females to follow a healthy lifestyle as a part of the management plan.

Almost two-thirds of participants had depression, anxiety, and stress. PCOS is known to increase mental health disorders [6,14,15]. The 2023 International Evidence-Based Guideline for the Assessment and Management of Polycystic Ovary Syndrome recommended screening females with PCOS for anxiety and depression using regionally validated screening tools [2]. Both depression and stress were associated with age and were more common in young individuals in the age group of 15-25. A similar finding was reported in Taiwan, and the authors suggested that irregular menses, obesity, and hyperandrogenism are more likely to frustrate younger females [26]. Depression, anxiety, and stress were associated with marital status and increased among divorced females. A study on Omani females with PCOS reported they receive divorce threats, which increases their psychological stress [19]. We did not explore the cause of divorce and cannot relate it to infertility, but it remains a possibility. Another possibility is the psychological burden of divorce itself [27] added on top of the PCOS burden.

Interestingly, individuals on diet and lifestyle modification were more likely to be depressed, anxious, and stressed compared to those who did not follow lifestyle modification advice. Lifestyle modifications reportedly have a positive effect on the mental health of people with various disorders [26,28]. This negative association should be explored in future studies. High BMI was associated with depression. Similar findings are reported from other parts of the globe [6,16].

The findings of this study should be interpreted in the light of some limitations, the main being the use of a non-probability sampling technique. Another limitation is dependence on self-reported PCOS diagnosis. Nonetheless, we asked individuals to state the tests used in the diagnosis to ensure authenticity. The voluntary nature of participation limits the possibility of false claims.

## Conclusions

PCOS is a common disorder in Saudi Arabia. It is associated with being in the age group of 26-35, being divorced, and having a positive family history of PCOS. More than two-thirds of females with PCOS had depression, anxiety, and stress. Young age was associated with depression and anxiety. Divorce and the use of diet and lifestyle modifications were associated with anxiety, depression, and stress. High BMI was associated with depression but not anxiety and stress.

## Figures and Tables

**Figure 2:**
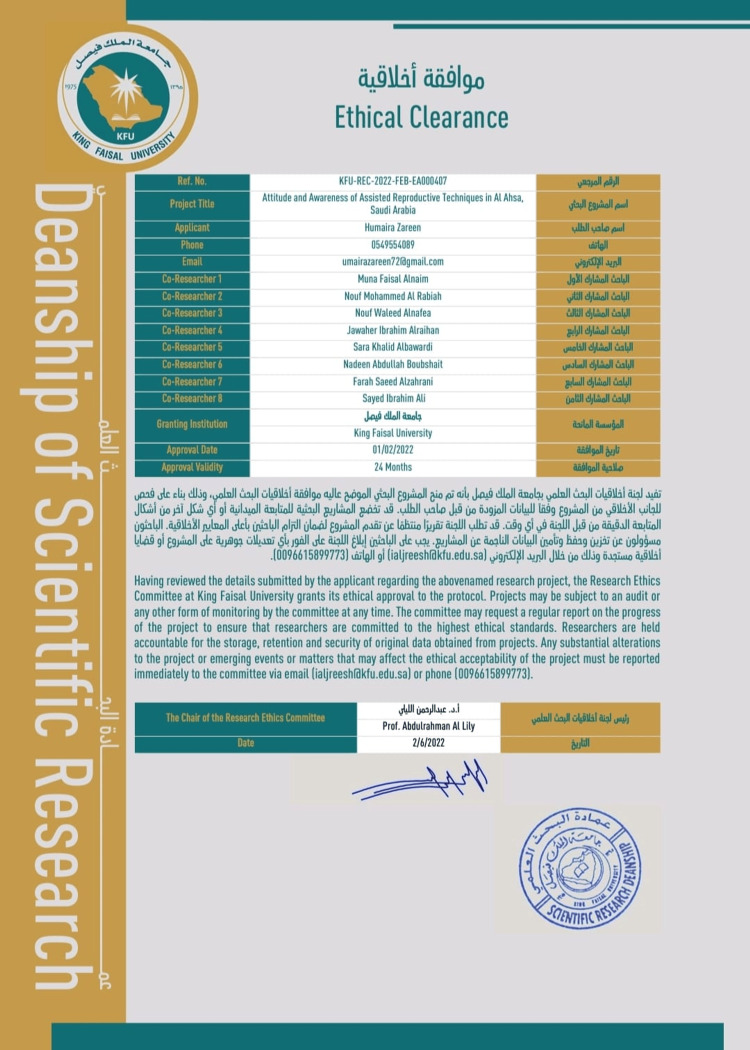
Ethical approval letter
